# Phylogenomic Reconstruction and Functional Divergence of the PARP Gene Family Illuminate Its Role in Plant Terrestrialization

**DOI:** 10.3390/ijms27010117

**Published:** 2025-12-22

**Authors:** Kun Yi, Qilin Yang, Zhen Ding, Daoyuan Zhang, Yan Wang, Bei Gao

**Affiliations:** 1 Xinjiang Key Laboratory of Biological Resources and Genetic Engineering, College of Life Science and Technology, Xinjiang University, Urumqi 830046, China; zillion_echo@163.com; 2 State Key Laboratory of Ecological Safety and Sustainable Development in Arid Lands, Xinjiang Institute of Ecology and Geography, Chinese Academy of Sciences, Urumqi 830011, China; yangqilin22@mails.ucas.ac.cn (Q.Y.); dingzhen22@mails.ucas.ac.cn (Z.D.); zhangdy@ms.xjb.ac.cn (D.Z.); 3 China-Tajikistan Belt and Road Joint Laboratory on Biodiversity Conservation and Sustainable Use and Xinjiang Key Laboratory of Conservation and Utilization of Plant Gene Resources, Xinjiang Institute of Ecology and Geography, Chinese Academy of Sciences, Urumqi 830011, China

**Keywords:** PARP, evolutionary history, DNA damage repair, bryophyte

## Abstract

The evolution of robust DNA repair mechanisms was a prerequisite for the conquest of land by plants, a transition that exposed them to harsh new environmental stressors. The poly (ADP-ribose) polymerase (PARP) family is central to this adaptation, as it orchestrates DNA repair and stress signaling pathways essential for coping with the elevated UV radiation and desiccation of terrestrial environments. Yet its early evolutionary origins are unknown. Here, we present a comprehensive reconstruction of the PARP family’s history across the plant kingdom. Our phylogenomic analysis reveals that *PARP* evolution ignited during the bryophyte radiation, expanding from a single ancestral algal gene into three distinct subfamilies (*PARP1*, *PARP2*, and *PARP3*). This diversification was driven by structural innovations in DNA-binding domains and a rewiring of transcriptional networks to respond to terrestrial challenges. We provide direct experimental support for this hypothesis through functional analysis of PARPs from the extremotolerant moss *Syntrichia caninervis*. We show that its PARP proteins provide multifaceted protection against UV radiation, heat, and genotoxic agents, and that recently duplicated *PARP2* genes are already diverging in function. Our work pinpoints the molecular adaptations in a key DNA repair family that enabled the greening of Earth and uncovers novel genetic targets for enhancing crop resilience.

## 1. Introduction

Poly (ADP-ribose) polymerase (PARP) possesses poly (ADP-ribose)ylation (PARylation) modification activity [1]. This enzymatic activity enables PARP and its target proteins to recruit DNA damage repair-associated factors while also conferring the ability to stabilize DNA replication forks and modify chromatin structure [2,3,4]. PARP attaches multiple, even hundreds of ADP-ribose units to target proteins in a NAD^+^-dependent manner [5,6,7]. This function relies on the PARP catalytic domain, a structure termed the PARP tag, which serves as a key identifier for members of the *PARP* gene family [8]. It is well known that the primary function of PARP is DNA damage repair, and this function is highly conserved across both plants and animals [9,10,11,12]. PARP enzymes function as critical sensors of genomic stress. They rapidly detect DNA lesions, such as single-strand breaks (SSBs) and double-strand breaks (DSBs), and facilitate repair by recruiting specific repair complexes to the damage site [2,13,14,15,16]. Beyond direct repair, PARP activity is also integrated with cell cycle checkpoints, helping to pause the cell cycle to ensure that repair is completed before division proceeds [17,18,19]. In *Arabidopsis thaliana*, *AtPARP1* and *AtPARP2* function in DNA damage signaling and repair by modifying DNA through ADP-ribosylation of terminal phosphate residues [20]. Undoubtedly, *PARP* genes constitute a crucial class of DNA damage repair genes, participating in multiple repair pathways following DNA damage.

In plants, PARP function extends beyond DNA repair to encompass a broad spectrum of stress responses, contributing to plant tolerance against various biotic and abiotic challenges [21,22,23,24]. For instance, PARP activity has been linked to plant-bacterial interactions in *A*. *thaliana* [25], responses to ionizing radiation in *Phaseolus vulgaris* and *A. thaliana* [23,26], seed viability in *A. thaliana* [27], and even developmental processes like gametophyte development in *Oryza sativa* (rice) [28]. Despite these insights into the functional diversity of PARP in higher plants, the deep evolutionary history and functional trajectory of this gene family, particularly in early plant lineages, remain largely unexplored.

Early investigations into plant PARPs primarily focused on a limited number of species, such as *A*. *thaliana* and *Zea mays*, and proposed classification systems that were not fully comprehensive [29]. With the advent of high-throughput sequencing and advanced bioinformatics tools, *PARP* gene family members have been identified across a wide range of plant species, primarily within the angiosperm lineage, including dicots (e.g., *A. thaliana*, *Nicotiana tabacum*) and monocots (e.g., *Zea mays*, *O. sativa*) [28,29,30,31]. This surge in genomic data now enables robust, multi-species analysis to reconstruct the evolutionary trajectory of the *PARP* gene family. While the expansion of the *PARP* family has been correlated with traits like lifespan in woody plants [32], a systematic exploration of its evolutionary history from algae to seed plants, encompassing its origin, diversification, and the establishment of functional roles, is still lacking. Critically, bryophytes (mosses), which represent a pivotal evolutionary transition to terrestrial life, have been largely overlooked in these studies. The colonization of land was not merely a change in habitat but a confrontation with a hostile environment. Early land plants were suddenly exposed to direct solar UV radiation, drastic temperature fluctuations, and periodic desiccation. These stressors were largely buffered in aquatic settings but represent potent sources of DNA damage and oxidative stress, necessitating the evolution of robust repair mechanisms [33]. Extremotolerant mosses like *Syntrichia caninervis* have evolved remarkable resilience to conditions such as extreme radiation and desiccation, making them ideal models to study the adaptive evolution of stress-response genes like *PARP*.

Therefore, to elucidate the deep evolutionary history of this vital gene family, we conducted a comprehensive phylogenomic and structural analysis of *PARP* genes across 175 plant species, from algae to angiosperms. Our study aimed to reconstruct the origin, expansion, and functional divergence of the *PARP* subfamilies, with a particular focus on their evolution during the key transition to land. We hypothesized that the transition from aquatic to terrestrial environments imposed selective pressures that drove the expansion and diversification of the *PARP* gene family, particularly during the emergence of bryophytes. Furthermore, we postulated that the PARP proteins from early land plants, such as the extremotolerant moss *S. caninervis*, would exhibit broad-spectrum tolerance to the specific abiotic stresses encountered during land colonization. To test these hypotheses, we conducted a comprehensive phylogenomic and structural analysis of *PARP* genes across 175 plant species and validated the functional predictions by characterizing *PARP* members from *S. caninervis* using a yeast heterologous expression system.

## 2. Results

### 2.1. Identification and Phylogenetic Analysis of the PARP Gene Family

**Figure 1:**
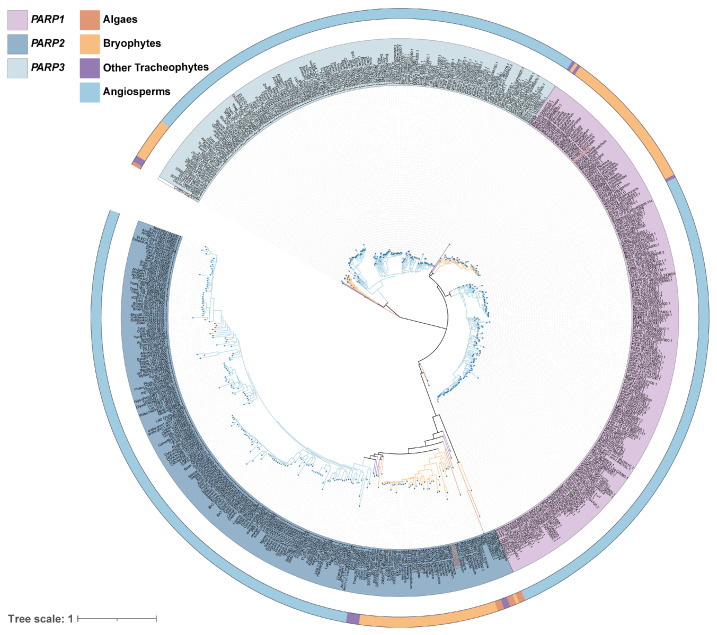
Phylogenetic relationships of 577 PARP proteins from 175 plant species. The maximum likelihood tree shows three distinct clades (PARP1, PARP2, PARP3), annotated by the inner color ring. The outer ring indicates the taxonomic group of each sequence.

A total of 577 PARP protein sequences were identified from 175 species using a combination of Blastp and HMMsearch methods. The final multiple sequence alignment comprised 468 amino acid residues. Following multiple sequence alignment, a phylogenetic tree was constructed using the JTT + F + R7 model, which was determined to be the optimal model for the dataset (Figure 1). The phylogenetic analysis robustly partitioned the PARP family into three distinct clades with high statistical confidence: PARP1 (Bootstrap Support = 60%), PARP2 (Bootstrap Support = 94%), and PARP3 (Bootstrap Support = 100%)**.** The PARP1 and PARP2 clades formed a monophyletic group, which is sister to the PARP3 clade.

Notably, PARP2 members were ubiquitously present across all analyzed species, from algae to angiosperms. In contrast, PARP1 and PARP3 members were absent in algae and some early land plants; for instance, the *S. caninervis* genome lacks a PARP3 homolog. Further analysis revealed that the charophyte alga *Chara braunii* possesses not only a PARP2 homolog but also a unique PARP protein (CHBRA95g00810) that formed a separate, basal branch distinct from the three main clades, potentially representing an ancient lineage. In the genome of our focal species, *S. caninervis*, we identified one *PARP1* gene and two *PARP2* genes. These two *PARP2* genes, which we designated *ScPARP2A* and *ScPARP2B*, are located in tandem on the same chromosome.

### 2.2. Expansion of the PARP Gene Family During Plant Terrestrialization

**Figure 2:**
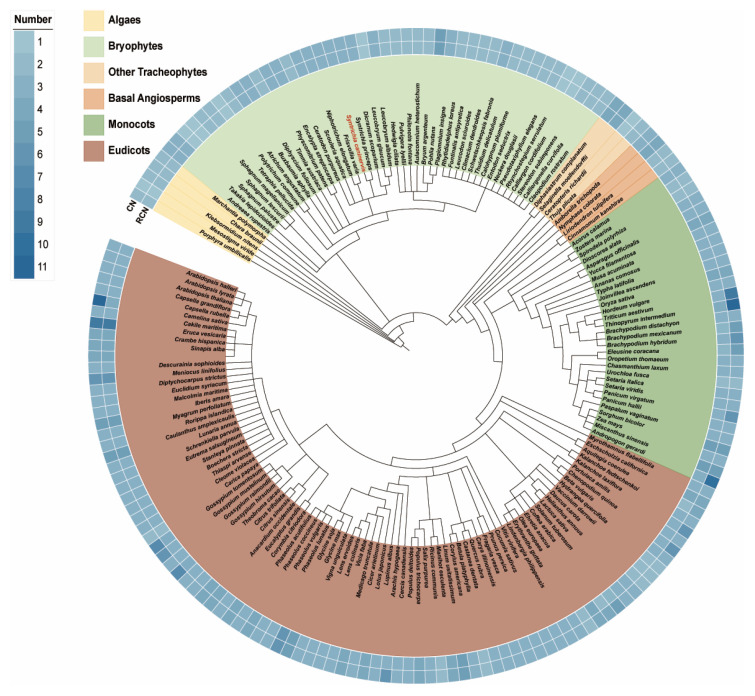
Copy number variation in the *PARP* gene family across the plant kingdom. The species tree shows the phylogenetic relationships among 175 species. The heatmap on them indicates the absolute copy number (CN) of *PARP* genes for each species. Major plant lineages are indicated by colored bars.

Analysis of *PARP* gene copy number (CN) across 175 species revealed a clear evolutionary trend of gene family expansion, particularly during the transition from aquatic to terrestrial life (Figure 2). In algal lineages, the *PARP* family is small and variably present; CNs ranged from zero to two, with the gene being entirely absent in several surveyed species. A notable expansion occurred in bryophytes, which typically harbored two or three *PARP* genes. This increase often resulted from duplications within the *PARP2* subfamily, as observed in species like *S. caninervis* and *Pohlia nutans*, and coincided with the emergence of the *PARP1* and *PARP3* subfamilies. Following this initial expansion, the *PARP* gene family size became remarkably conserved in tracheophytes (vascular plants). Most diploid vascular plants possess three *PARP* genes—one from each of the *PARP1*, *PARP2*, and *PARP3* clades. This conservation is further highlighted by the relative copy number (RCN), which normalizes for ploidy. While absolute CNs vary in polyploid species, the RCN in most tracheophytes is consistently three, suggesting strong selective pressure to maintain one functional copy from each major subfamily after land colonization.

### 2.3. Subfamily Specific Conserved Motifs and Gene Structures

**Figure 3:**
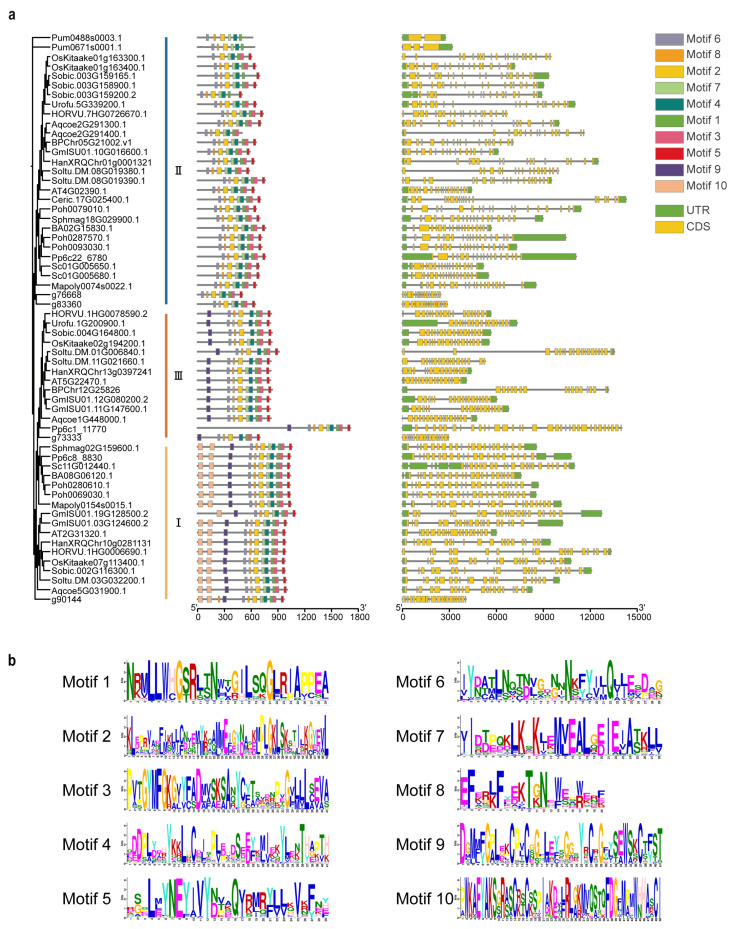
Structural and phylogenetic analysis of the *PARP* gene family. (**a**) The phylogenetic tree (left) categorizes PARP proteins into three subfamilies (I, II, III). Aligned with the tree, the conserved motif distribution (middle, colored boxes) and exon-intron structure (right) are shown for each corresponding gene. (**b**) Sequence logos detailing the conserved amino acid residues for each of the ten motifs.

To investigate the structural evolution of the *PARP* gene family, we analyzed the conserved protein motifs and gene structures of *PARP* members from 19 representative species (Figure 3). The analysis of conserved motifs revealed both shared and subfamily specific patterns that align perfectly with the phylogenetic classification. A core set of motifs, including Motif 5 (annotated as the PARP catalytic domain), was present in nearly all PARP proteins, confirming their shared ancestry.

However, distinct motif compositions clearly differentiate the three subfamilies. The PARP1 subfamily is characterized by the presence of a full complement of 10 motifs, including the unique Motif 10, which corresponds to the DNA-Ligase Zn-finger domain essential for DNA damage recognition. The PARP3 subfamily contains 9 motifs, sharing Motif 9 (PADR1 domain) with PARP1 but lacking Motif 10. In contrast, the PARP2 subfamily exhibits the simplest motif architecture, typically containing only 7–8 motifs and lacking both Motif 9 and Motif 10. These subfamily specific motif architectures suggest early functional divergence following gene duplication.

Analysis of gene structure revealed that *PARP* genes in land plants are structurally complex, typically comprising over ten exons. This is in stark contrast to the *PARP* gene from the alga *P. umbilicalis*, which has a much simpler structure. This trend towards increased structural complexity, through the gain or elongation of introns, appears to coincide with the territorialization of plants and the functional diversification of the *PARP* family.

### 2.4. Synteny Analysis Reveals Divergent Evolutionary Fates of PARP Subfamilies

**Figure 4:**
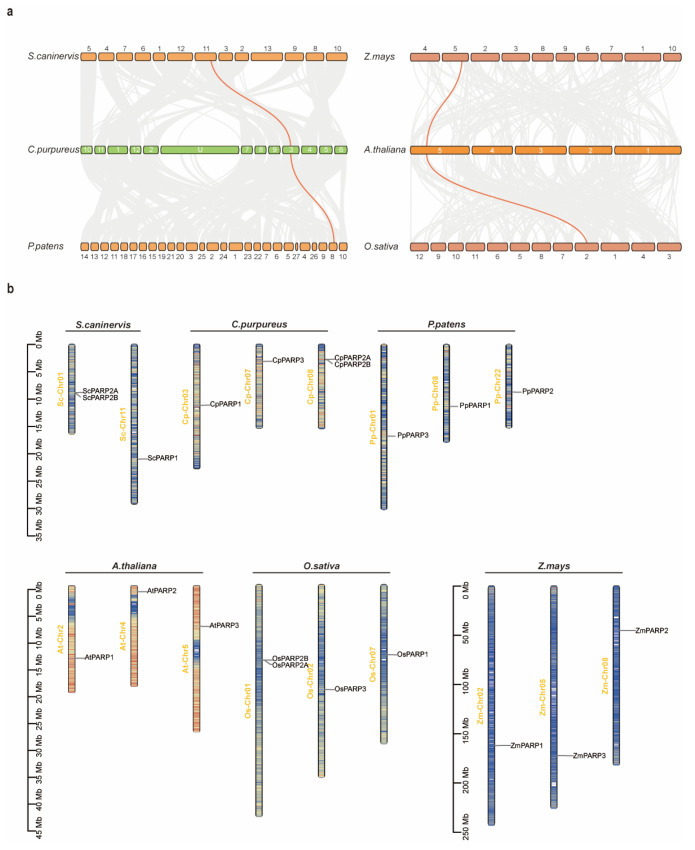
Syntenic relationships and chromosomal locations of *PARP* genes. (**a**) Comparative synteny analysis within bryophytes (*S. caninervis*, *C. purpureus*, *P. patens*) and angiosperms (*Z. mays*, *A. thaliana*, *O. sativa*). Gray lines connect syntenic blocks across genomes, while orange lines specifically highlight the conserved syntenic relationships for *PARP1* and *PARP3* genes. (**b**) Chromosomal maps showing the physical location of *PARP* genes in the six species.

To trace the evolutionary origins and duplication mechanisms of *PARP* genes, we conducted a comparative synteny analysis across key bryophyte and angiosperm species, representing the early stages of land colonization and highly evolved vascular plants, respectively (Figure 4a). The analysis revealed distinct evolutionary trajectories for the different *PARP* subfamilies. We identified conserved syntenic blocks surrounding the *PARP1* locus between *C. purpureus* and both *P. patens* and *S. caninervis*. Similarly, the *PARP3* locus was found in syntenic regions between *A. thaliana* and the two monocots, *O. sativa* and *Z. mays*. These findings suggest that the *PARP1* and *PARP3* genes have been retained in conserved genomic locations following large-scale duplication events within their respective lineages.

In striking contrast, no syntenic relationships were detected for any *PARP2* genes among the species analyzed. Instead, the expansion of the *PARP2* subfamily appears to be driven by local, small-scale duplications. This is strongly supported by the chromosomal localization data (Figure 4b), which shows that the two *PARP2* copies in both *S. caninervis* and *O. sativa* are arranged in tandem on the same chromosome (*Sc*-Chr01 and *Os*-Chr01, respectively). Overall, *PARP* genes from different subfamilies are located on separate chromosomes, and no large-scale synteny was observed between the bryophyte and angiosperm groups, reflecting their deep evolutionary divergence.

### 2.5. Sequence Divergence and Conservation Among PARP Subfamilies

**Figure 5:**
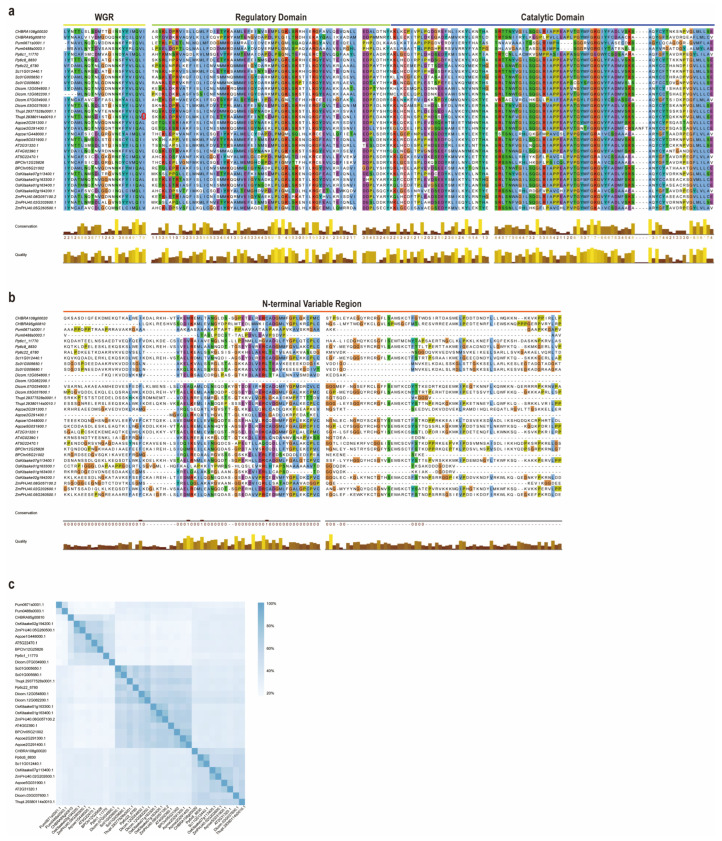
Multiple sequence alignment and similarity analysis of representative PARP proteins. (**a**) Alignment of the conserved C-terminal region, encompassing the PARP regulatory and catalytic domains (Displayed above the comparison matrix, respectively). The histogram below the alignment indicates the conservation level at each position. (**b**) Alignment of the highly divergent N-terminal region, showing extensive gaps and low sequence homology. (**c**) A heatmap representing the pairwise sequence identity matrix for the 32 PARP proteins. The color intensity corresponds to the percentage of sequence identity, as indicated by the color key.

To explore the molecular basis of PARP functional diversification, we performed a detailed sequence analysis of 32 representative PARP proteins selected to cover major plant lineages, including algae, bryophytes, lycophytes, gymnosperms, and angiosperms (Figure 5). Detailed physicochemical properties of these proteins, including molecular weight, isoelectric point, and predicted subcellular localization, are summarized in Table 1. Multiple sequence alignment revealed a striking contrast between the C-terminal and N-terminal regions of these proteins. The C-terminal region, which encompasses the PARP regulatory and catalytic domains, is highly conserved across all analyzed species and subfamilies (Figure 5a). This high degree of conservation underscores the fundamental enzymatic mechanism shared by all PARP family members.

In sharp contrast, the N-terminal regions are characterized by significant divergence, featuring extensive gaps (insertions/deletions) and low sequence homology (Figure 5b). These variable N-terminal regions are known to harbor domains critical for specific functions, such as DNA binding and protein–protein interactions (e.g., SAP and PADR1 domains). The pronounced sequence divergence in this area strongly suggests that functional specialization among the PARP subfamilies is primarily driven by the evolution of their N-termini.

To quantify the overall sequence relationships, we generated a pairwise identity matrix (Figure 5c). This analysis confirmed the distinct separation of the three main PARP subfamilies, with intra-subfamily identities generally high (>60%) while inter-subfamily identities were significantly lower (<40%). The matrix also highlights the unique evolutionary position of algal proteins from *P. umbilicalis* and *C. braunii*, which exhibit low identity to all land plant PARP subfamilies, consistent with their basal phylogenetic placement.

### 2.6. Evolutionary Diversification of Regulatory Elements in PARP Promoters

**Figure 6:**
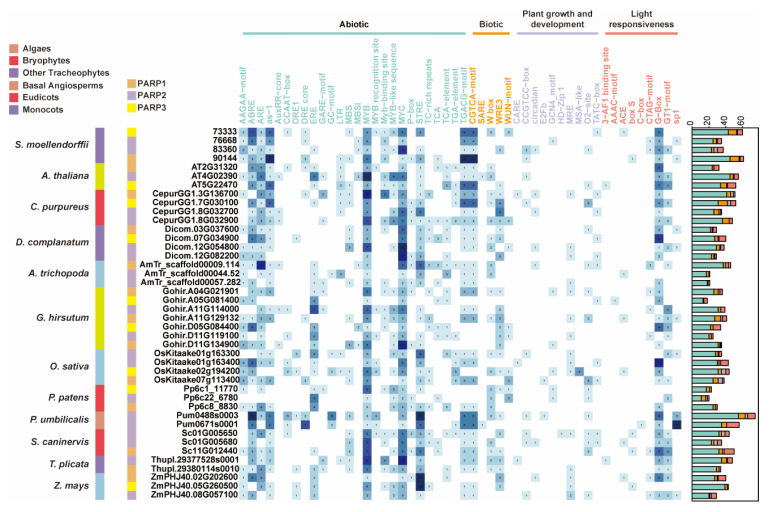
Distribution of *cis*-acting regulatory elements in the promoters of *PARP* genes. The heatmap illustrates the abundance of various cis-elements across selected *PARP* gene promoters. The color intensity of each cell corresponds to the count of a specific element, with darker blue indicating a higher number. Elements are categorized into functional groups: Abiotic, Biotic, Plant growth and development, and Light responsiveness. The vertical color bar on the left indicates the *PARP* subfamily (*PARP1*, *PARP2*, *PARP3*) for each gene. The stacked bar chart on the right summarizes the total count of cis-elements for each promoter, color-coded by the functional categories.

To understand the evolution of *PARP* gene regulation, we analyzed the cis-acting regulatory elements in the promoter regions of *PARP* genes from representative species spanning the evolutionary spectrum from algae to angiosperms (Figure 6). The analysis revealed that *PARP* promoters are densely populated with a wide array of cis-elements, predominantly those associated with abiotic stress and light responsiveness, suggesting that transcriptional regulation is a key layer of PARP functional control.

A clear evolutionary trend was observed from aquatic algae to land plants. The promoter of the algal *P. umbilicalis PARP* gene contains a basal set of stress-related elements but is notably poor in light-responsive elements. In contrast, bryophyte promoters, particularly those from *S. caninervis*, show a marked increase in both the diversity and abundance of elements. This includes a significant enrichment of light-responsive elements (e.g., G-box, 3-AF1 binding site) and the appearance of novel elements absent in algae, such as the MRE element, which is critical for light-regulated expression. Furthermore, the salicylic acid-responsive TCA element, a key component in biotic stress signaling, emerges in *S. caninervis* and becomes prevalent in higher plants.

When analyzed by subfamily, distinct regulatory signatures become apparent. For instance, the promoters of *PARP1* genes across various species consistently show a high abundance of MYB and MYC binding sites, which are central to stress and developmental regulation. The promoters of *PARP3* genes are enriched in TCA-elements, hinting at a specialized role in biotic stress responses. These distinct cis-element architectures among subfamilies suggest that the functional divergence of *PARP* genes was accompanied by significant rewiring of their transcriptional regulatory networks.

### 2.7. Predicted 3D Structures Reveal Subfamily Specific Conformations and Divergence in Tandem Duplicates

**Figure 7:**
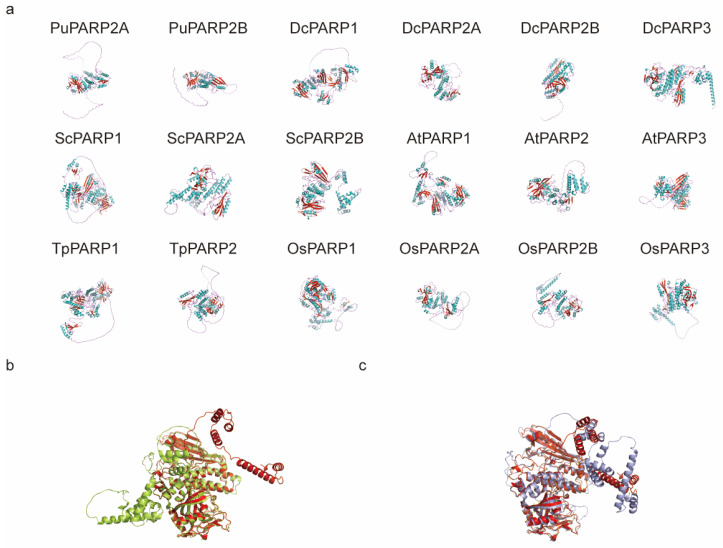
Predicted three-dimensional structures of plant PARP proteins. (**a**) Gallery of predicted 3D structures for 18 PARP proteins from six species: *P. umbilicalis* (Pu), *S. caninervis* (Sc), *D. complanatum* (Dc), *A. thaliana* (At), *T. plicata* (Tp), and *O. sativa* (Os). Secondary structures are colored (α-helices in cyan, β-sheets in red). (**b**) Structural alignment of the tandem duplicates from *S. caninervis*, ScPARP2A (red) and ScPARP2B (green), highlighting differences in peripheral loop conformations. (**c**) Superposition of *S. caninervis* PARP2 paralogs (*Sc*PARP2A in red, *Sc*PARP2B in blue) onto the structure of *A. thaliana At*PARP2 (light gray/transparent), revealing varying degrees of structural conservation and divergence.

To investigate the structural basis of PARP function, we predicted the three-dimensional structures of 18 PARP proteins from six representative species covering major plant lineages using AlphaFold (Figure 7a). The predicted models consistently show a conserved catalytic core rich in α-helices and β-sheets. However, significant variations were observed in the conformation and length of peripheral loops and terminal regions, particularly among the three subfamilies. For instance, in *A. thaliana*, both *At*PARP1 and *At*PARP2 feature prominent, flexible N-terminal extensions, creating an ‘open’ conformation that may be important for DNA or substrate binding. In contrast, *At*PARP3 adopts a more ‘closed’ and compact structure, consistent with its reported lack of enzymatic activity.

We then focused on the tandemly duplicated genes *ScPARP2A* and *ScPARP2B* from *S. caninervis* to explore potential structural divergence following local duplication. While both proteins share a highly similar core fold, their peripheral structures, especially the surface-exposed loops, exhibit notable differences (Figure 7b). To place these structures in an evolutionary context, we aligned them with the well-characterized *At*PARP2 from *A. thaliana*. The structural alignment shows that while both *Sc*PARP2 paralogs are broadly similar to *At*PARP2, *Sc*PARP2B displays more significant conformational deviations in several regions compared to *Sc*PARP2A (Figure 7c). This structural divergence suggests that *Sc*PARP2A and *Sc*PARP2B may have undergone sub- or neo-functionalization after their duplication.

### 2.8. S. caninervis PARP Proteins Confer Multi-Stress Tolerance in Yeast

**Figure 8:**
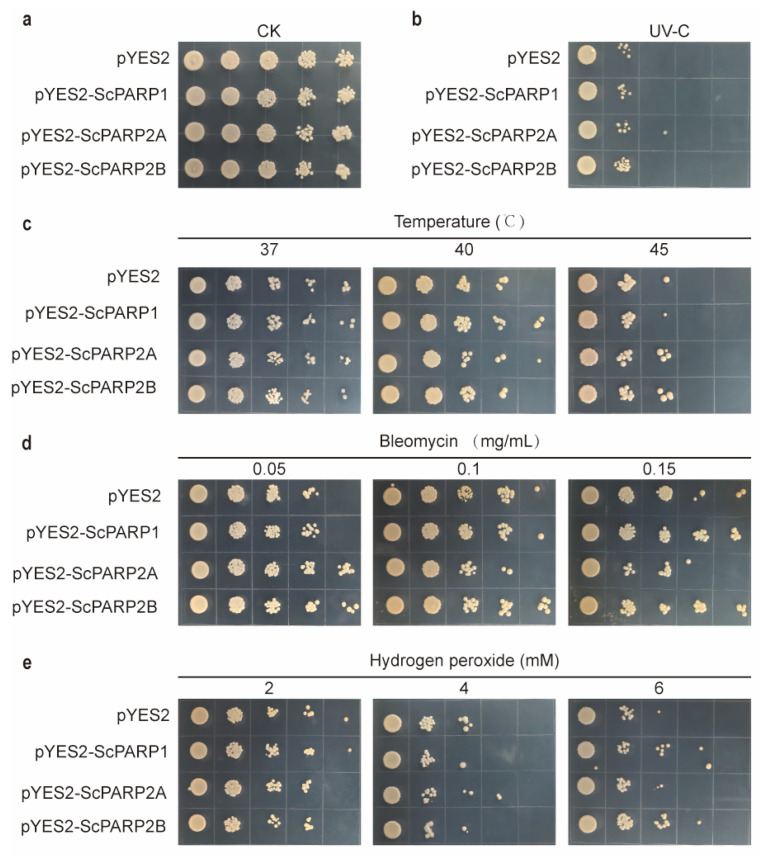
Functional analysis of *S. caninervis PARP* genes in yeast stress tolerance assays. Yeast strains transformed with an empty pYES2 vector (control) or vectors expressing *ScPARP1*, *ScPARP2A*, or *ScPARP2B* were serially diluted and spotted onto solid medium. (**a**) Growth under non-stress control conditions (30 °C). (**b**) Growth after exposure to UV radiation. (**c**) Growth under heat stress at the indicated temperatures. (**d**) Growth on medium containing different concentrations of the DNA-damaging agent bleomycin. (**e**) Growth on medium containing different concentrations of the oxidizing agent H_2_O_2_.

**Figure 9:**
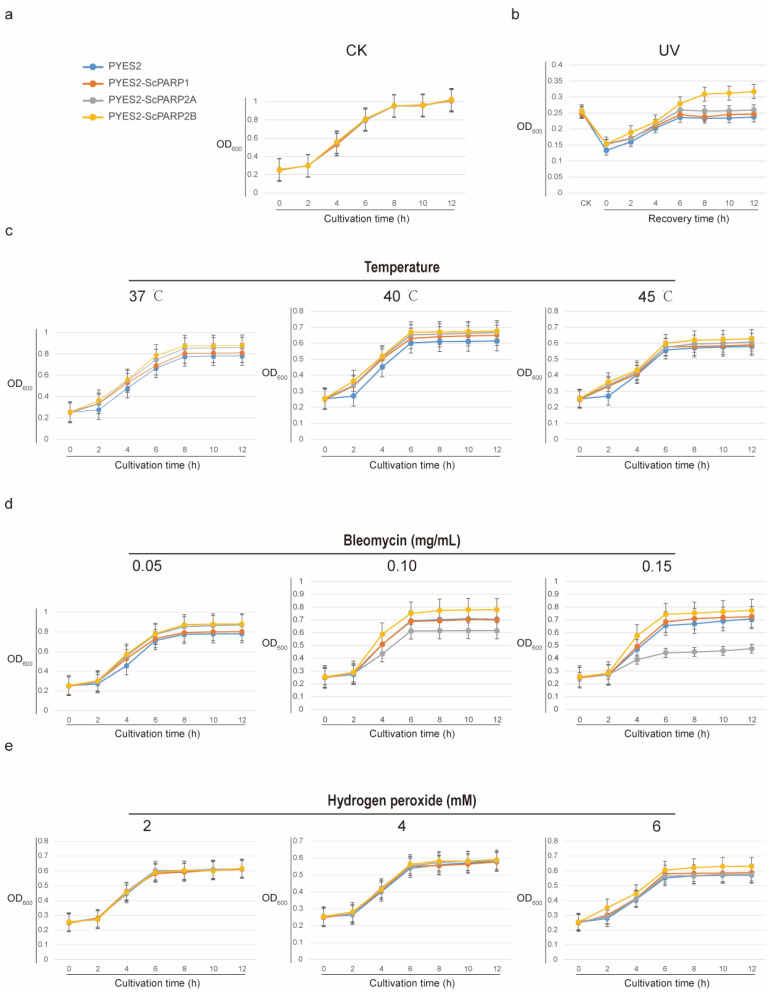
Growth Curve of *ScPARP*-Transgenic Yeast in Stress Resistance Experiments. Yeast strains transfected with the empty vector pYES2 (control) or vectors expressing *ScPARP1*, *ScPARP2A*, and *ScPARP2B* were cultured under various stress conditions, with OD_600_ measurements taken every 2 h. (**a**) Growth curves under non-stress control conditions (30 °C). (**b**) Growth curve after UV irradiation, where CK represents pre-treatment. 0 h denotes the first measurement post-treatment. (**c**) Growth curve under heat stress at specified temperatures. (**d**) Growth curve in media containing varying concentrations of the DNA-damaging agent bleomycin. (**e**) Growth curve in media containing varying concentrations of the oxidizing agent H_2_O_2_.

To functionally characterize the *PARP* genes from *S. caninervis*, we expressed *ScPARP1*, *ScPARP2A*, and *ScPARP2B* in yeast and assessed their ability to confer stress tolerance using spot dilution assays. First, under optimal growth conditions (30 °C), all yeast strains, including the empty vector control, grew robustly and showed no discernible differences (Figure 8a and Figure 9a). This confirms that the heterologous expression of the *ScPARP* genes is not detrimental to yeast growth, providing a valid baseline for stress experiments. Next, we evaluated the tolerance to DNA damage induced by UV radiation. Following UV exposure, all strains exhibited significant growth inhibition. However, the strain expressing *ScPARP2B* displayed markedly better survival and recovery compared to the control, as well as to the strains expressing *ScPARP1* and *ScPARP2A* (Figure 8b and Figure 9b), suggesting a specialized role for *ScPARP2B* in responding to UV-induced damage.

We then tested the response to heat stress at 37 °C, 40 °C, and 45 °C. Compared to the control strain exhibiting certain growth defects at high temperatures, all three strains expressing the *ScPARP* gene demonstrated a degree of thermotolerance (Figure 8c). Combined with the growth curves (Figure 9c), it can be observed that at all three temperatures, the three strains expressing the *ScPARP* gene showed superior growth capacity compared to the control strain during the 0–4 h period, with this advantage being particularly pronounced at 45 °C. In media containing varying concentrations of bleomycin, yeast expressing *ScPARP2B* also demonstrated superior performance compared to the other two strains. *ScPARP2A* exhibited even poorer growth activity (Figure 8d and Figure 9d). This suggests that the tandem repeat genes *ScPARP2A* and *ScPARP2B* of *S. caninervis* may possess functional differences. Finally, we assessed tolerance to oxidative stress using hydrogen peroxide (H_2_O_2_). Unlike other stress factors, the expression of the three *ScPARP* genes does not appear to confer a clear advantage (Figure 8e). In the growth curves under these conditions, we also observed that only yeast expressing *ScPARP2B* under 6 mM H_2_O_2_ conditions exhibited a certain degree of recovery and growth advantage, while the other two strains showed no significant advantage (Figure 9e). This may also suggest a slight advantage of *ScPARP2B* over the other two.

Collectively, these assays reveal that the *S. caninervis* PARP proteins provide broad protection against diverse environmental and genotoxic stresses, with evidence of functional specialization, particularly for *ScPARP2B* in UV tolerance.

## 3. Discussion

This study provides the first comprehensive reconstruction of the *PARP* gene family’s evolutionary history across the plant kingdom. By integrating phylogenomic, structural, and functional analyses, we reveal that the family’s diversification was not gradual but occurred in a major burst coinciding with the territorialization of plants, establishing a function that was critical for adaptation to life on land. Our findings strongly support a model where the plant *PARP* family evolved from a single, ancestral *PARP2*-like gene present in algae. This conclusion is supported by three lines of evidence from our study. First, our phylogenetic analysis places the PARP2 clade at the most basal position of the plant PARP tree (Figure 1). Second, PARP2 is the only subfamily found in early-diverging algal lineages, whereas PARP1 and PARP3 are conspicuously absent (Figure 2). Third, PARP2 proteins possess the simplest motif architecture, lacking key domains present in the other two subfamilies (Figure 3). The PARP proteins in algae like *P. umbilicalis* lack domains associated with efficient DNA damage recognition (e.g., the Zn-finger corresponding to Motif 10), suggesting that these primordial PARPs may have possessed basic catalytic activity but lacked the sophisticated sensory functions of their land plant counterparts. The unique, deeply branching PARP homolog in the charophyte *Chara braunii* may represent an ancient lineage or an early evolutionary experiment that did not persist, underscoring the dynamic nature of gene family evolution during this early period.

Based on evolutionary relationships, the 577 PARP proteins were grouped into three major categories (Figure 1), consistent with the classification of *A. thaliana* PARP: PARP1, PARP2, and PARP3 [34]. By comparing conserved motifs and protein sequence conservation matrices, we arrive at the same conclusion. Clearly, the classification of *PARPs* in plants is remarkably simple, with far fewer categories than those found in mammals [35]. Green algae occupy a pivotal position in plant evolution and are considered the ancestors of all green plants [36,37]. Therefore, they represent a crucial node in the study of gene evolutionary history. When identifying members of the *PARP* gene family through homology alignment, we detected relevant members only in a few algal species, while most algae lacked them (Figure 2). *C. braunii* is a charophyte belonging to an early group in plant evolutionary history, representing the origin of terrestrial plants [38]. In this study, we identified a unique *PARP* member, CHBRA95g00810, which does not belong to any of the three major branches. Given the evolutionary position of *C*. *braunii*, we speculate that this gene may represent one of the early origin genes within the *PARP* gene family. Based on species classification and phylogenetic tree analysis, all species in the constructed tree contain members of the *PARP2*, while the other two species categories are absent in algae. This suggests that *PARP2* genes most likely originated in algae and represent the earliest-emerging *PARP* class. Analysis of conserved motifs in the 19 representative species we selected reveals that *PARP2* genes exhibit relatively few conserved motif categories, consistent with the hypothesis that *PARP2* represents an early-originating class. Referencing the phylogenetic tree topology, we propose that both *PARP1* and *PARP3* genes likely originated from *PARP2* genes. This evolutionary scenario mirrors that of other key plant gene families, such as the phytochromes, where gene duplication events provided the raw material for the functional innovations required for terrestrial adaptation [39]. Furthermore, *PARP1* and *PARP3* first appeared in moss plants, indicating that the differentiation of these two *PARP* genes occurred during the moss phase, representing a terrestrial origin. However, not all moss plants possess all three *PARP* classes simultaneously; some mosses contain only *PARP1* and *PARP2* members. Therefore, we speculate that the differentiation of *PARP1* likely occurred prior to that of *PARP3*. In summary, *PARP* genes emerged as early as the algae stage, initially giving rise to *PARP2* genes. Subsequently, during the moss stage, the two additional *PARP1* and *PARP3* gene classes diverged.

After completing the identification of the *PARP* gene family, we conducted a statistical analysis of its members (Figure 2). Following the direction of plant evolution, we observed a progressive expansion of the *PARP* gene family along the evolutionary trajectory, suggesting a potential diversification of its functions [40]. First, in higher tracheophytes, the relative copy number remained highly conserved across nearly all species, consistently comprising three members—one per class. Based on this, we speculate that *PARP* gene functions may have stabilized during the tracheophyte evolution. In contrast, during earlier evolutionary stages, the relative copy number of the *PARP* gene family showed significant variation, evolving from an initial zero members to three members. Thus, the evolutionary peak of the *PARP* gene family occurred during the evolution of algae and mosses. Mosses occupy a crucial transitional position in the evolutionary history of plants and are considered early terrestrial species [41]. During the landing process, multiple new environmental stresses, such as high-intensity UV radiation, desiccation, and extreme temperature fluctuations [42,43]. These intensifying pressures likely demanded that the *PARP* gene family possess additional functions to maintain genomic stability. Subcellular localization indicates the site where a protein functions within the cell and can, to some extent, reflect its biological role [44]. Thus, the function of the *PARP* gene with diverse subcellular localization should also exhibit multifunctionality, although the predictive results require further validation (Table 1). This study also identified numerous tandemly repeated *PARP2* genes, which are widely distributed across multiple species, such as *S. caninervis* and *O. sativa*. Regarding colinearity, the *PARP2* gene did not show colinearity between bryophytes and angiosperms. Furthermore, even within the same genus, no colinearity was observed between the *PARP* genes of *C. purpureus* and *S. caninervis*. These results indicate that *PARP2* genes underwent intense evolutionary changes, particularly during the moss stage (Figure 4). In summary, we propose that during the evolution of plants from algae to tracheophytes, *PARP* genes may have retained replication copies to cope with increasingly complex environments. These additional copies likely diverged into distinct functions during evolution.

The PARP catalytic domain and PARP regulatory domain are two important conserved domains within the PARP family, primarily located at the C-terminal end of PARP proteins [8,12,45]. In protein 3D structure prediction, distinct PARP proteins all possess a prominent core structure corresponding to two major conserved domains (Figure 7). Notably, the two PARP proteins in the algal *P. umbilicalis* lack Motif 5, whereas other PARPs possess it. Since Motif 5 belongs to the PARP catalytic domain, we speculate that Motif 5, which emerged after algae, endows PARP with broader functional capabilities (Figure 3). Simultaneously, certain regions of PARP proteins exhibit open conformations, suggesting inherent flexibility that enables binding to specific ligands or protein partners. Differences between PARP proteins primarily reside in their external structures. While these structural variations do not compromise core functionality, they imply the existence of sub-functionalization.

The DNA-binding domain of PARP proteins is located at their N-terminus [46,47]. We found that the N-termini of various PARP proteins exhibit significant differences and contain numerous gap regions (Figure 5). As early as 1998, scientists proposed that higher plant PARPs exhibit two distinct patterns: those possessing two N-terminal DNA-ligase Zn-finger regions and those lacking them [29]. The N-terminal zinc finger structure of PARP functions as a DNA nick sensor and serves as a crucial regulatory component in the cellular response to DNA damage [45]. This study reveals that PARP1 possesses Motif 10, distinct from the other two PARPs, and this motif is precisely located within the domain (Figure 3). This structure is not exclusive to higher plants but is widely present in PARP1 of bryophytes. Based on this, we hypothesize that among the three PARP enzymes, PARP1 may exhibit superior recognition and be binding to DNA damage sites, thereby possessing stronger DNA damage repair capabilities. This conjecture aligns with Gu’s related research, which indicates that AtPARP1 is the primary PARP protein responding to DNA damage [27]. Compared to PARP2, PARP1 and PARP3 possess an additional motif 9, which belongs to the PADR 1 domain segment (Figure 3). PADR1 is considered the third zinc-binding domain of PARP proteins and participates in coordinating protein–protein interactions [48]. Additionally, the N-terminal differences in PARP are also reflected in the SAP domain. The SAP domain has been demonstrated to be essential for specific DNA-binding activity [49]. The two PARP proteins in the alga *P. umbilicalis* lack any of the three domains, but we found that one of *C. braunii* PARP2 proteins (CHBRA108g00020) shares structural features with PARP1. It contains not only the PADR1 domain but also two zinc finger structures for DNA binding. This finding suggests that PARP may have begun acquiring DNA-binding and DNA damage signaling capabilities during the *C. braunii* phase, with the domain characteristics of PARP1 emerging as early as the algal era. In *A. thaliana*, AtPARP3 likely loses its DNA-binding capacity precisely because it lacks both the SAP domain and the DNA-ligase Zn-finger region. Its three-dimensional structure also indicates weaker open conformation, thereby resulting in the absence of PARP enzymatic activity in AtPARP3 [27]. Similarly, PARP3 in other angiosperms exhibit the same pattern. Most PARP2 proteins possess an SRA domain but lack PADR1, whereas PARP1 proteins contain two N-terminal zinc finger structures and PADR1. This may explain why PARP1 and PARP2, despite both possessing DNA damage repair capabilities, exhibit distinct modification patterns [20].

The cis-elements of the *PARP* gene contain numerous components associated with non-biotic stress responses and light responses, reflecting the potential role of this gene family in stress response and light regulation (Figure 6). Based on the general changes in the cis-acting elements of *PARP* during plant evolution, we have made certain inferences about the functional shifts within the *PARP* gene family. In the early stages, the functions of *PARP* in algae were relatively limited, primarily associated with abiotic stress responses. Mosses are considered transitional groups representing the early stages of plant colonization, during which plants faced environmental impacts such as drought and increased light exposure [50,51,52]. Therefore, in mosses, *PARP* genes began to exhibit diverse stress response elements and light response elements. Light response elements MRE and the salicylic acid response element TCA started appearing in the promoter regions of *PARP* genes in *S.caninervis* and higher tracheophytes, indicating that *ScPARP* genes had already begun evolving toward those of higher tracheophytes. Furthermore, all three *ScPARP* genes contain numerous MYB and MYC elements, which are key regulatory sites for plant stress resistance. This suggests *ScPARP* plays a functional role in stress response mechanisms.

In conclusion, our study systematically charts the evolutionary journey of the plant *PARP* gene family from a simple algal ancestor to a functionally diverse essential for terrestrial life. Future research should focus on dissecting the specific protein interaction networks and enzymatic substrates of each *PARP* subfamily to fully unravel their distinct biological roles. The potent stress-tolerant *PARP* genes identified in *S. caninervis* also represent promising candidates for genetic engineering to enhance stress resilience in crops, opening a new avenue for translating evolutionary insights into agricultural applications.

## 4. Materials and Methods

### 4.1. Identification Statistics and Physicochemical Property Predictions for Members of the Multispecies PARP Gene Family

This study used the protein sequence of AtPARP2 (AT4G02390.1) as the query sequence. Members of the *PARP* gene family were identified and screened using the Blastp algorithm against the Phytozome v14 (https://phytozome-next.jgi.doe.gov/, accessed on 16 December 2024) (E-value ≤ 1 × 10^−5^), the Dry but not Dead database (DWD, http://desiccation.novogene.com/, accessed on 1 December 2024), and the 1K Plant Transcriptome Project (1KP) database (https://db.cngb.org/onekp/, accessed on 8 December 2024). These searches covered 175 species spanning algae, mosses, gymnosperms, basal angiosperms, dicotyledons, monocotyledons, and other tracheophytes. Due to excessive sequence redundancy in the 1KP database, we filtered redundant sequences with similarity >90% using CD-HIT v4.8.1 (parameters: -c 0.9 -n 5) [53,54]. Simultaneously, we integrated representative algal genome data from the NCBI database with the high-quality T2T genome of *S. caninervis* assembled by our group [55,56]. Using the same query sequences, we performed localized Blastp searches via TBtools v2.330 [57]. Finally, all candidate protein sequences were validated using the hmmsearch module of HMMER 3.3.2 [58]. Sequences containing both the PARP regulatory domain (PF00644) and the PARP catalytic domain (PF02877) were selected (E-value < 1 × 10^−10^) [59]. This process yielded PARP protein sequences from 175 species, and representative PARP protein sequences from selected plants were further extracted. The protein sequences obtained from these identifications have been compiled in Table S2.

Subsequently, based on species ploidy data (integrated from Phytozome and literature), we quantified the copy number (CN) and relative copy number (RCN = CN × 2/ploidy) of *PARP* genes across species. We have also compiled these statistics into a table located in Table S3. We constructed a species phylogenetic tree using the AVI algorithm within the Taxonomy module of the NCBI database (https://www.ncbi.nlm.nih.gov/taxonomy, accessed on 1 January 2025). The copy number heatmap, optimized and integrated using the iTOL platform (https://itol.embl.de/, accessed on 1 January 2025), enabled simultaneous visualization of phylogenetic relationships and copy number variation. Additionally, protein data for PARP family members from *Porphyra umbilicalis*, *P. patens*, *S. caninervis*, *Ceratodon purpureus*, *A. thaliana*, *O. sativa*, and *Z. mays* were predicted using the Expasy online platform (https://www.expasy.org/, accessed on 20 May 2025), and their subcellular localization information was predicted using the WoLF PSORT online tool (https://www.genscript.com/tools/wolf-psort, accessed on 21 May 2025).

### 4.2. Phylogenetic Tree Construction and Conserved Motifs

The 577 identified PARP protein sequences were aligned using Muscle v5.3 [60], and the resulting alignment was trimmed using TrimAL v1.5 [61]. A maximum likelihood (ML) phylogenetic tree was constructed using IQ-TREE 2 v2.0.7 [62]. The best-fit substitution model was automatically selected by ModelFinder, and branch support was assessed with 1000 ultrafast bootstrap replicates. The final tree was visualized and annotated using the iTOL platform. Sequence alignments for selected species were visualized using Jalview v2.11.5.0 [63]. Conserved motifs in representative PARP proteins were identified using the MEME suite (https://meme-suite.org/meme/, 27 April 2025) with the following parameters: maximum number of motifs set to 10, and motif width between 6 and 50 amino acids. The results, alongside gene structures, were visualized using the Gene Structure View tool in TBtools v2.330 [57].

### 4.3. Chromosome Distribution and Colinearity Analysis

First, we obtained the relevant genomic files for *P. umbilicalis*, *P. patens*, *C. purpureus*, *A. thaliana*, *O. sativa*, and *Z. mays* from the Phytozome, Ensembl Plants (https://plants.ensembl.org/index.html, 1 June 2025), and DWD databases, and integrated them with the *S. caninervis*-T2T genomic file from our group. Using the “One step MCScanX” tool in TBtools v2.330 with default parameters, we analyzed the collinearity among these species [57]. Subsequently, we utilized the “Gene Location Visualization from GTF/GFF” module in TBtools v2.330 to map the chromosomal distribution of *PARP* gene family members across the aforementioned species and performed visual modifications [57]

### 4.4. Cis-Element Analysis

The 2-kilobase upstream sequences of *PARP* genes were extracted from the Phytozome database for 19 representative species selected to cover key evolutionary nodes from algae to angiosperms (specific species are listed in Figure 6). These included *P. umbilicalis*, *P. patens*, *C. purpureus*, *D. complanatum*, *A. thaliana*, and *O. sativa*. For *S. caninervis*, the sequences were extracted using the “GTF/GFF3 Sequences Extract” module in TBtools v2.330 [57]. Subsequently, these sequences were submitted to the PlantCARE (https://bioinformatics.psb.ugent.be/webtools/plantcare/html/, 15 August 2025) for cis-acting element prediction and analysis. After obtaining the cis-element data, we performed preliminary screening of the results and counted the number of cis-elements associated with abiotic stress, biotic stress, light exposure, or growth and development. Following this, we visualized the data using the ggplot2 package in Rstudio [64].

### 4.5. Three-Dimensional Structure Prediction and Comparison

Using the Alpha-Fold online (alphafoldserver.com), we performed three-dimensional structure predictions for all PARP proteins from six plant species chosen to represent the major evolutionary lineages: *P. umbilicalis* (algae), *S. caninervis* (bryophytes), *D. complanatum* (lycophytes), *T. plicata* (gymnosperms), *A. thaliana* (dicots), and *O. sativa* (monocots) [65]. Structural alignments and superposition of selected protein models were also performed within PyMOL v3.1.0. to compare their conformational similarities and differences [66].

### 4.6. Preliminary Functional Characterization of the ScPARP Gene in a Yeast System

The coding sequences of *ScPARP1*, *ScPARP2A*, and *ScPARP2B* were amplified from *S. caninervis* cDNA and cloned into the pYES2-eGFP vector via homologous recombination. The resulting constructs and the empty vector (as a control) were transformed into the *Saccharomyces cerevisiae* strain W303-1A using the lithium acetate method. Successful transformation and gene expression upon galactose induction were confirmed by colony PCR and reverse transcription PCR (RT-PCR), respectively. Primer sequences are listed in Supplementary Table S1. For stress tolerance assays, yeast strains were cultured overnight in SD-Ura selective liquid medium, then transferred to SG-Ura liquid medium for 12 h to induce protein expression. The induced cultures were adjusted to an OD_600_ of 0.4, and then a 10-fold serial dilution series was prepared. Five microliters of each dilution were spotted onto SG-Ura solid medium plates containing different stressors. Heat stress: Plates were incubated at 37 °C, 40 °C, and 45 °C for 48–72 h. Oxidative stress: Plates contained 2, 4, or 6 mM H_2_O_2_ and were incubated at 30 °C. DNA damage stress: Plates contained 0.05, 0.10, or 0.15 mg/mL bleomycin and were incubated at 30 °C. UV stress: Spotted plates were exposed to UV-C radiation at a dosage of 400 μW/cm^2^ using a UV crosslinker, then incubated at 30 °C in the dark for 0.5 h. A control plate without any stress treatment was incubated at 30 °C. All experiments were repeated three times, and representative results are shown.

Simultaneously, we measured and plotted the growth curves of yeast under the aforementioned conditions. First, we adjusted the four transgenic yeast strains to an OD600 of 0.25. The yeast was cultured in liquid SG-Ura medium containing various stress factors. All stress conditions are consistent with those described above. Set up three biological replicates for each condition. Measure their OD_600_ values at 0, 2, 4, 6, 8, 10, and 12 h, and plot growth curves.

## 5. Conclusions

This study provides a comprehensive evolutionary history of the plant *PARP* gene family. Our phylogenetic analysis demonstrates that the family originated in algae with an ancestral *PARP2*-like gene. Following the colonization of land, a major diversification occurred during the bryophyte stage, giving rise to the *PARP1* and subsequently the *PARP3* subfamilies. This expansion coincided with a burst of functional innovation, driven by the divergence of N-terminal domains and the acquisition of complex regulatory networks responsive to terrestrial stresses like UV radiation and dehydration. Functional assays in the key bryophyte *S. caninervis* confirmed the multifaceted stress-tolerance functions of its PARP members and provided direct evidence for the sub-functionalization of duplicated *PARP2* genes. Our findings pinpoint the transition to land as the critical period that shaped the functional landscape of this vital DNA repair gene family in plants.

## Figures and Tables

**Table 1:** Physicochemical Properties and Subcellular Localization Prediction of PARP Protein.

Gene Model ID	Species	Class	CDS Length (bp)	Peptide Residue (AA)	MW	Aliphatic Index	PI	Gravy	Predicted Subcellular Localization
CHBRA108g00020	*C*. *braunii*	II	3270	1089	121,577.68	71.84	8.73	−0.652	nucl
CHBRA95g00810	*C*. *braunii*	II	4116	1371	151,524.42	67.39	5.68	−0.694	nucl
Pum0671s0001.1	*P*. *umbilicalis*	II	1914	637	66,498.18	82.35	9.56	−0.101	chlo
Pum0488s0003.1	*P*. *umbilicalis*	II	1848	615	63,911.07	87.54	5.14	0.024	cyto
Pp6c8_8830	*P*. *patens*	I	3126	1041	116,706.17	70.93	8.72	−0.648	chlo
Pp6c22_6780	*P*. *patens*	II	2286	761	85,951.74	77.27	6.65	−0.691	cyto
Pp6c1_11770	*P*. *patens*	III	5136	1711	188,430.86	73.30	6.04	−0.476	nucl
Sc11G012440.1	*S*. *caninervis*	I	3105	1034	116,281.36	69.63	8.55	−0.710	chlo
Sc01G005650.1	*S*. *caninervis*	II	2067	688	77,372.43	74.11	5.49	−0.688	nucl
Sc01G005680.1	*S*. *caninervis*	II	2076	691	78,442.27	75.15	6.81	−0.630	nucl
Dicom.03G037600.1	*D*. *complanatum*	I	3144	1047	117,999.89	77.03	8.5	−0.549	chlo
Dicom.12G054800.1	*D*. *complanatum*	II	1845	614	69,747.59	81.30	7.20	−0.527	cyto
Dicom.12G082200.1	*D*. *complanatum*	II	1410	469	53,469.98	71.13	8.38	−0.574	cyto
Dicom.07G034900.1	*D*. *complanatum*	III	2340	779	89,289.41	79.24	5.43	−0.508	cyto
Thupl.29380114s0010.1	*T*. *plicata*	I	3030	1009	115,201.4	76.12	8.6	−0.72	nucl
Thupl.29377528s0001.1	*T*. *plicata*	II	2160	719	81,838.94	77.36	7.54	−0.641	cyto
Aqcoe5G031900.1	*A*. *coerulea*	I	3015	1004	112,424.41	73.87	8.79	−0.587	nucl
Aqcoe2G291300.1	*A*. *coerulea*	II	2130	709	80,559.18	80.28	8.62	−0.572	nucl
Aqcoe2G291400.1	*A*. *coerulea*	II	1491	496	56,318.7	83.73	7.88	−0.329	vacu
Aqcoe1G448000.1	*A*. *coerulea*	III	2466	821	92,095.57	76.15	5.35	−0.514	cyto
AT2G31320.1	*A*. *thaliana*	I	2952	983	111,233	71.63	8.8	−0.645	nucl
AT4G02390.1	*A*. *thaliana*	II	1914	637	72,175.67	77.27	5.92	−0.602	nucl
AT5G22470.1	*A*. *thaliana*	III	2448	815	91,534.01	76.54	5.14	−0.514	nucl
OsKitaake07g113400.1	*O*. *sativa*	I	2934	977	110,155.92	76.05	8.85	−0.585	nucl
OsKitaake01g163300.1	*O*. *sativa*	II	1818	605	67,742.34	78.07	7.88	−0.405	cyto
OsKitaake01g163400.1	*O*. *sativa*	II	1959	652	73,333.98	74.66	6.89	−0.613	cyto
OsKitaake02g194200.1	*O*. *sativa*	III	2496	831	92,403.84	73.9	5.44	−0.522	cyto
ZmPHJ40.02G202600.1	*Z*. *mays*	I	2943	980	110,475.33	75.84	8.74	−0.562	chlo
ZmPHJ40.08G057100.2	*Z*. *mays*	II	1962	653	73,113.11	78.85	8.57	−0.535	cyto
ZmPHJ40.05G260500.1	*Z*. *mays*	III	2511	836	93,243.73	73.49	5.12	−0.49	nucl

## Data Availability

The original contributions presented in this study are included in the article/Supplementary Material. Further inquiries can be directed to the corresponding author(s).

## Supplementary Materials

The following supporting information can be downloaded at: https://www.mdpi.com/article/10.3390/ijms27010117/s1.
