# Supplementary material for: Phylogenomic Reconstruction and Functional Divergence of the PARP Gene Family Illuminate Its Role in Plant Terrestrialization
Source: Int J Mol Sci. 2025 Dec 22;27(1):117. doi: 10.3390/ijms27010117 (PMC12785305; doi:10.3390/ijms27010117)
Supplement: Supplementary file 1 [file ijms-27-00117-s001.zip › Table S1.pdf]

Table S1 Primer name and sequence

Primer Name	Sequence
ScPARP1(SacI)-F	attaagcttggtaccgagctcATGGCGACTCCACCAGTGC
ScPARP1(BamHI)-R	ttctcccttacccatggatccCCGTTTGTGCTTGAAGTTAACCT
ScPARP2A(SacI)-F	attaagcttggtaccgagctcATGTCTAACAAACTCACTGTGGGC
ScPARP2A(BamHI)-R	ttctcccttacccatggatccTTTGTAATTGAACCTCACTTGTAGAAGA
ScPARP2B(SacI)-F	attaagcttggtaccgagctcATGTCCAATAAACTGACTGTCGGC
ScPARP2B(BamHI)-R	ttctcccttacccatggatccGTAACTGAACCGCACTTGTAGAAGA
PCR-ScPARP1-F	ATGGCGACTCCACCAGT
PCR-ScPARP1-R	GAAGTTAACCTTGAGCAAGAACC
PCR-ScPARP2A-F	ATGTCTAACAAACTCACTGTGG
PCR-ScPARP2A-R	ACCTCACTTGTAGAAGATAGCG
PCR-ScPARP2B-F	GTCCAATAAACTGACTGTCGGC
PCR-ScPARP2B-R	GTCCAATAAACTGACTGTCGGC
qPCR-ScPARP1-F	GGTTGAGGCAGCTGGAGG
qPCR-ScPARP1-R	AGGCACTCCCCTGGCATA
qPCR-ScPARP2A-F	CAATCGGCTTGATGCGGC
qPCR-ScPARP2A-R	CGTCCACCTTGACACGCT
qPCR-ScPARP2B-F	CAACAGAGCCTTGCCGGA
qPCR-ScPARP2B-R	GCAGTCCGGATGCTCCAC
